# Exploring defensive medicine: examples, underlying and contextual factors, and potential strategies - a qualitative study

**DOI:** 10.1186/s12910-023-00949-2

**Published:** 2023-10-10

**Authors:** Mohammad Hossein Eftekhari, Alireza Parsapoor, Ayat Ahmadi, Neda Yavari, Bagher Larijani, Ehsan Shamsi Gooshki

**Affiliations:** 1 Medical Ethics and History of Medicine Research Center, Tehran University of Medical Sciences, Tehran, Iran; 2 Knowledge Utilization Research Center, Tehran University of Medical Sciences, Tehran, Iran; 3 Department of Medical Ethics, School of Medicine, Isfahan University of Medical Sciences, Isfahan, Iran; 4 Endocrinology and Metabolism Research Institute, Tehran University of Medical Sciences, Tehran, Iran; 5 Monash Bioethics Center, Monash University, Melbourne, Australia

**Keywords:** Defensive medicine, Medical ethics, Medical law, Iran

## Abstract

**Background:**

Medical errors, unsatisfactory outcomes, or treatment complications often prompt patient complaints about healthcare providers. In response, physicians may adopt defensive practices to mitigate objections, avoid complaints, and navigate lengthy trial processes or other potential threats. However, such defensive medicine (DM) practices can carry risks, including potential harm to patients and the imposition of unnecessary costs on both patients and the healthcare system. Moreover, these practices may run counter to accepted ethical standards in medicine.

**Methods:**

This qualitative study involved conducting semi-structured interviews with 43 physicians, among whom 38 were faculty members at medical universities, 42 had administrative experience at various levels of the health system, and 23 had previously served as health system policymakers. On average, the participants had approximately 23.5 years of clinical experience. The selection of participants was based on purposive sampling. Data collection through interviews continued until data saturation was achieved.

**Results:**

Based on the findings, DM manifests in both positive and negative forms, illustrated by instances like ordering unnecessary lab tests, imaging, or consultations, reluctance to admit high-risk patients, and avoiding high-risk procedures. The study participants identified a range of underlying and contextual factors contributing to DM, encompassing organizational-managerial, social, personal, and factors inherent to the nature of defensive medical practices. The results also highlight proposed strategies to address and prevent DM, which can be grouped into organizational-managerial, social, and those focused on modifying the medical complaints management system.

**Conclusion:**

DM is a multifaceted and significant phenomenon that necessitates a comprehensive understanding of its various aspects, including interconnected and complex structures and underlying and contextual factors. While the results of this study offer a solid foundation for informing policy decisions within the healthcare system and include some explanatory policy suggestions, we encourage policymakers to complement the findings of this study with other available evidence to address any potential limitations and to gain a more comprehensive understanding of the policymaking process related to DM.

**Supplementary Information:**

The online version contains supplementary material available at 10.1186/s12910-023-00949-2.

## Introduction

 Enhanced understanding and awareness of diseases, coupled with remarkable advancements in health technologies, have significantly impacted the conventional patient-physician relationship. Consequently, patients and their families now harbor heightened expectations from physicians [1]. While the medical and scientific communities have made substantial progress, it is noteworthy that the prevalence of medical complaints has surged in developed as well as developing nations, including Iran [2–4]. One of the repercussions resulting from the surge in malpractice lawsuits against physicians is the adoption of defensive behavior. Defensive behavior in medical interventions, commonly known as DM, encompasses actions taken by physicians that lack medical necessity and offer no benefits to the patient (positive DM). It also includes abstaining from high-risk procedures that are medically warranted and could benefit the patient (negative DM). The primary motivation behind such behavior is to evade potential adverse consequences, particularly legal lawsuits. Regrettably, both forms of DM are increasingly becoming commonplace in the medical profession, leading to escalated healthcare costs, and occasionally compromising the quality of services rendered. For instance, subjecting patients to unnecessary invasive diagnostic tests not only exposes them to additional risks but also incurs additional expenses [5, 6]. As an example, the perception of a heightened risk of malpractice lawsuits among gynecologists has led to a significant increase in defensive behavior, particularly in the form of defensive C-Sects. [7–9]. As a consequence, as one cross-sectional study showed in Brazil, specialists are opting for C-sections six times more frequently to avoid potential complaints related to normal vaginal deliveries [10]. It is crucial to note that available evidence indicates that surgeons, gynecologists, and obstetricians face the highest rate of liability claims [11]. DM has primarily been the subject of study in the United States and European countries. In these regions, commonly observed forms of DM involve the implementation of unnecessary diagnostic and therapeutic measures. These practices include conducting unwarranted invasive procedures, excessive hospitalizations, and the over-documentation of medical records [12]. While defensive interventions can be observed across all medical specialties, they are particularly prevalent in certain fields characterized by inherently higher risk profiles. Notably, specialties such as obstetrics and gynecology, neurosurgery, and orthopedic surgery exhibit a greater frequency of defensive practices [12].

There exists a wide array of defensive behaviors among physicians, and the prevalence of these behaviors varies across countries and specialties. Conversely, the prevalence of DM is influenced by various contextual factors [13, 14]. DM shares close similarities with other aspects of medical practice. For instance, positive defensive behaviors can be seen as instances of unnecessary services or overuse driven by defensive intentions. From an ethical perspective, at least in many situations, DM raises concerns as prescribing or performing diagnostic or therapeutic procedures solely for defensive purposes may contradict physicians’ ethical commitment to their patients and society. This approach could potentially violate medical ethics principles and values, including prioritizing beneficence, respecting the right to informed decision-making and consent, and upholding equity [15]. The potential negative impacts of DM on patients and the healthcare system are manifold. They include unjustifiably burdening the health system with costs, limiting access to healthcare services, causing harm to patients, compromising patient safety and undermining the overall quality of care [6, 16–18].

Although a few studies in Iran have indicated a high prevalence of DM [19, 20], there is a notable lack of evidence regarding the various aspects of this phenomenon, such as its types, examples and contextual factors. The unique characteristics of the Iranian health system may differentiate it from other systems, underscoring the importance of conducting research in this area through a qualitative approach. The practice of DM is intricately influenced by healthcare professionals’ clinical judgment and experience. It should be acknowledged that it extends beyond merely deviating from correct practice; instead, it is often a conscientious response driven by the complexities inherent in individual cases and the clinical environment. Understanding this nuanced aspect is crucial for a comprehensive examination of DM’ multifaceted nature. This feature of DM justifies our qualitative approach in the present study to provide a better understanding of the related issues.

## Materials and methods

A qualitative study was undertaken after obtaining ethical clearance from the Research Ethics Committee of Tehran University of Medical Sciences (IR.TUMS.MEDICINE.REC.1399.731). Semi-structured interviews were conducted with a total of 43 physicians, and the interviews were concluded when data saturation was achieved. Among the participants, 38 were faculty members of medical universities, 42 had administrative experience at various levels of the health system, and 23 had served as health system policymakers for a significant duration. The mean clinical experience of the participants was 23.5 years. The selection of participants was carried out through purposive sampling. For more information about the characteristics of the participants, please refer to Table 1.

**Table 1:** Participants’ characteristics

Specialty/Subspecialty	Sex	University Faculty member	Work experience in the public sector (years)	Work experience in the private sector (years)	Clinical Practice Experience (years)	Healthcare Facility Management experience (years)	Health Policymaking experience (years)
Emergency medicine specialist	Male	Yes	Yes	2	22	10	NO
Gynecologist	Female	Yes	Yes	Yes	25	15	NO
General practitioner	Male	NO	NO	28	29	16	7
Internist	Male	Yes	Yes	7	20	30	12
Pediatrician, Subspecialist in pediatric nephrology	Male	Yes	Yes	NO	25	19	15
Pediatrician	Male	Yes	Yes	Yes	35	30	20
Anesthesiologist	Male	Yes	Yes	Yes	16	6	NO
Ophthalmologist, Subspecialist in oculoplastic surgery	Male	Yes	Yes	Yes	19	5.5	NO
Internist, Rheumatology subspecialist	Female	Yes	Yes	1	18	2	NO
Orthopedic surgeon	Male	Yes	Yes	4	20	6	2
General practitioner	Male	NO	Yes	NO	26	20	_ NO
Gynecologist	Female	NO	Yes	4	21	20	NO
Gynecologist, Subspecialty fellowship in Gynecologic Oncology	Female	Yes	Yes	25	33	15	NO
Gynecologist, Subspecialty fellowship in Maternal-fetal medicine	Female	Yes	Yes	Yes	36	30	25
Medical ethicist, lawyer, and General practitioner (M.D. /Ph.D.)	Male	Yes	Yes	25	25	5	NO
Pediatrician, Subspecialist in pediatric endocrinology	Male	Yes	Yes	Yes	30	11	4
Orthopedic surgeon, Subspecialty fellowship in knee joint surgery	Male	Yes	Yes	NO	15	2	NO
Emergency medicine specialist	Male	Yes	Yes	NO	24	13	7
General practitioner	Male	NO	Yes	Yes	22	22	22
Anesthesiologist	Male	Yes	Yes	NO	15	5	NO
ENT specialist	Male	Yes	Yes	Yes	30	35	20
Anesthesiologist	Male	Yes	+ Yes	Yes	27	11	11
Anesthesiologist	Male	Yes	Yes	NO	23	5	-
Ophthalmologist, Retina Subspecialist	Male	Yes	Yes	Yes	25	10	NO
Anesthesiologist	Male	Yes	Yes	6	20	6	NO
General Surgeon	Male	Yes	Yes	20	30	26	30
General surgeon, plastic surgery subspecialist	Male	Yes	Yes	10	33	14	15
Dentist, Maxillofacial surgeon	Male	Yes	Yes	Yes	32	25	25
Neurosurgeon	Male	Yes	+ Yes	+ Yes	20	3	1
Internist, Subspecialist in cardiology	Male	Yes	26+	8	36	30	4
Ophthalmologist, Cornea Subspecialist	Female	Yes	Yes	4	20	11	NO
Internist, gastroenterology Subspecialist	Male	Yes	Yes	Yes	17	5	NO
Internist, endocrinology Subspecialist	Male	Yes	Yes	Yes	28	27	NO
Orthopedic surgeon, subspecialty fellowship in knee joint surgery	Male	+ Yes	Yes	NO	12	11	5
Internist, Rheumatology Subspecialist	Male	Yes	Yes	_ NO	5	NO	NO
General surgeon, Subspecialist in liver and bile duct surgery	Male	Yes	Yes	Yes	30	14	10
Anesthesiologist	Male	Yes	+ Yes	Yes	35	25	15
Cardiologist, Subspecialty fellowship in Interventional Cardiology	Male	Yes	Yes	10	16	5	NO
Medical ethicist and General practitioner (M.D. /Ph.D.)	Male	NO	20	3	20	12	4
General surgeon, Subspecialist in pediatric surgery	Female	Yes	Yes	----	20	20	16
Medical ethicist and General practitioner (M.D. /Ph.D.)	Male	Yes	Yes	Yes	6	7	2
Internist, Subspecialist in gastroenterology	Male	Yes	Yes	Yes	20	1	- NO
Anesthesiologist	Male	Yes	Yes	Yes	27	26	15

The interviewees comprised individuals with work experience in both governmental and private sectors. In adherence to research ethics principles, the study’s objectives and methodology were thoroughly explained to the participants. Prior to conducting the interviews, the confidentiality of the data obtained was assured, and all participants were informed about the strict confidentiality measures in place. To maintain confidentiality, all data collected from the interviews were stored securely in a locked computer, and access was limited only to the study investigators, who had special password-protected access. Furthermore, the information gathered will be securely deleted at the latest three years after the article’s publication.

Following a concise overview of the concept of DM, participants were asked a series of open-ended questions concerning the underlying and contextual factors, methods, and examples of DM, as well as potential strategies and solutions to confront it. The interview guide used for this purpose is provided as a Supplementary file in this paper. The study adhered to the ethical guidelines outlined in the General Ethical Guidance for Medical Research with Human Participants in the Islamic Republic of Iran [21] in all aspects of its implementation.

The interviews were conducted with the explicit consent of the participants, and each interview lasted between 20 and 70 min. Subsequently, every interview was transcribed verbatim to ensure accuracy and completeness. To enhance transcription quality, member checking was performed, wherein fifteen participants were invited to review and verify their own transcribed interviews. The next step involved a meticulous reading of the interview contents multiple times, followed by content analysis using the Framework Method analysis process. Framework analysis is a systematic approach utilized for describing and interpreting qualitative data. It involves creating a coherent framework that integrates themes derived from both inductive and deductive approaches. This process consists of five interconnected phases: Familiarization, Identifying a thematic framework, Indexing, Charting, and Mapping, and interpretation [22]. The interviews continued until data saturation was achieved, signifying that no added information was being provided by participants in the last three interviews. At this point, data collection was deemed sufficient for the study’s objectives.

## Results

The results extracted from the interviews were classified into three primary categories, encompassing underlying and contextual factors, examples of DM, and potential strategies to mitigate and prevent DM (Table 2). These 3 main categories and their 9 themes finally included 63, 33, and 57 codes respectively (Tables 3, 4 and 5).

**Table 2:** Summary of categories and themes

Main categories	Themes	Subthemes
Underlying and Contextual Factors of Defensive Medicine	Organizational-managerial factors	Factors related to medical education and training.
		Factors Related to the management of health facilities and health system policymaking
		Factors related to the medical complaints system
	Social factors	Factors related to the patient-physician relationship
		Factors related to the general culture of society
	Physicians’ personal factors	The poor motivation of physicians for risk-taking
		Physician’s Personality Traits and psychological condition
	Factors related to the nature of medicine and medical interventions	Factors related to the nature of medicine
		Factors related to the nature of the medical intervention
Examples of DM	Positive DM	-
	Negative DM	-
Suggested strategies to Reduce and Prevent Defensive Medicine	Modification of the medical complaints managing system	-
	Social strategies	-
	Organizational-managerial strategies	-

**Table 3:** Underlying and contextual factors of DM

Themes	Subthemes	Codes
Organizational-managerial factors	Factors related to medical education and training.	Lack of sufficient scientific and practical skills which would end in using several Para clinical interventions to prevent error
		Lack of officially approved national guidelines for disease diagnosis and treatment
		Insufficient training of physicians in evidence-based medicine, critical appraisal, and clinical reasoning
		Fading of ethical-oriented clinical relationship and bureaucratization and legalization of the physician-patient relationship
		The presence of some defensive considerations even in educational material and medical textbooks (e.g. recommendations of some emergency medicine textbooks for CT scans upon parents’ insistence despite lack of medical indications)
		Not allocating enough time to patient examination and clinical judgment, and using multiple Para clinical interventions to avoid error
		Performing defensive interventions as learned from superiors and professors
	Factors Related to the Management of health centers and health system policymaking	The dominance of a technology-centered attitude over medical practices has led to physicians’ concerns about malpractice in case of not using technology
		Physicians’ feeling of lack of sufficient legal and professional support from responsible organizations including professional organizations.
		A poor referral system and the inability of physicians to refer patients properly when they find themselves unqualified to treat them.
		Presence of defensive interventions as routine medical practice including diagnostic and treatment procedures in health care centers
		Lack of transparency in medical liability laws, regulations, and procedures
		Limitations of liability insurance coverage packages in forms such as setting time limits for coverage after each medical intervention.
		Insufficient sensitivity of public and private insurance companies about unnecessary prescriptions and practices including those with defensive motivations
		Facilitating filing complaints against physicians by related regulatory and supervising institutions
		Force and demand of Health facilities’ managers - (who could be liable as well as physicians in case of complaints and lawsuits) for using defensive measures.
		Lack of balance between the expectations of society and patients (which is high) on one hand and available resources and infrastructures (which are always limited)
		Lack of enough equipment and infrastructure in small health facilities, for management of possible adverse complications of medical practice such as single-specialty facilities or remote and rural health centers.
		High-demanding conditions for junior physicians and residents, beyond their practical and scientific capabilities
		Physicians’ lack of trust in the documentation system in healthcare centers
		Overcrowding of public health care facilities and inability to concentrate properly on diagnosis and treatment
		Equal diagnostic and therapeutic tariffs of high-risk and critically ill patients and normal patients which decreases the risk-taking
		The level of available equipment, resources, and support is higher in well-equipped referral university hospitals where physicians are trained and usually lower in smaller and less affluent centers where physicians would work after graduation.
		Reprimanding and blaming attitudes and presence of a culture of blame in clinical environments and fear of blame from colleagues
	Factors related to the medical complaints system	The strict approach of judicial authorities and supervising agencies regarding refraining from some procedures that are not indicated medically, and the indifference of courts and other complaint-handling organizations toward excess and unnecessary procedures
		Sharing the experience of being summoned to court with other colleagues, and physicians’ concerns arising from previous unpleasant experiences of colleagues about the process of trial in courts or other complaint-handling organizations
		Lack of uniform practice in handling medical complaints in different courts and other complaint-handling organizations which makes such organizations unpredictable for
		A multiplicity of medical complaint-managing organizations, including courts, the Medical Council, the Ministry of Health, and the Governmental Discretionary Punishments Organization (GDPO)
		Arbitrary judgment of complaint-handling authorities
		Concerns about criminal liability in medical malpractice cases
		Judicial authorities’ inattention to the systemic nature of medical errors while managing medical complaints and imposing most of the liabilities on physicians
		Lack of a system for primary assessment of complaints and summoning doctors for baseless, unjustified, and non-scientific complaints
		Concerns about the lack of enough respect for medical professionals and the human dignity of physicians whose cases are being investigated in complaint-handling organizations
		Physicians’ lack of knowledge about judicial proceedings and laws in courts and other complaint-handling organizations
Social factors	Factors related to the patient-physician relationship	Insufficient communication skills of physicians to interact with patients
		Presence of a paternalistic approach in the patient-physician relationship and a feeling that it is not necessary to obtain informed consent for every medical procedure (if informed consent is obtained, defensive interventions would be reduced ).
		Patients’ demands for and satisfaction with multiple Para clinical procedures
		Personality traits of some patients like being demanding, having hostile behavior, and being obsessive
		Concerns about being accused of scientific incompetence
	Factors related to the general culture of society	Increased public expectations from physicians due to a relative increase in health literacy and awareness of medical advances
		Dissemination of misinformation and pseudoscience, through cyberspace, and its effects on public expectations
		Encouraging patients to file complaints to lawyers
		The reduced general level of social capital in society
		Gradual familiarity of patients with their rights including their right for filing complaints from physicians
		Change of public religious beliefs and attitudes toward the role of medical interventions in the treatment of patients; In the past healing was seen more as something from God and the physician was merely a tool for healing, while increasingly physicians have a more significant role in this regard according to public beliefs.
		Increasing financial motives for filing a complaint against physicians to receive indemnity.
		Concerns about people’s hostile, threatening, or insulting confrontations with medical complications or malpractice, especially in small cities and rural and marginal areas.
		The increasing number of reports of social harassment against medical professionals. In the news and social media.
		Physicians are concerned about the negative consequences of medical complications in certain well-known and famous patients like politicians, celebrities, etc.
Physicians’ personal factors	The poor motivation of physicians for risk-taking	Believing in the unfairness of medical tariffs
		A feeling of weakening of mutual trust between patient and physician in society
		Lack of trust in the impartiality of the system of handling medical complaints
	Physician’s Personality Traits and psychological condition	Poor self-confidence due to a feeling of scientific or practical incompetence
		Personal traits such as conservative, obsessive, or histrionic personality which increase their need to receive assurance through involving colleagues in the treatment process by requesting a consultation, par clinical services, etc.
		Involving other physicians to share the responsibility and liability.
		Lack of mental preparedness of some physicians to cope with the stress of working in new circumstances
		Insufficient cultural competence of physicians when they start practice in diverse cultural contexts.
Factors related to the nature of medicine and medical interventions.	Factors related to the nature of medicine	Increasing trend of specialization and sub-specialization of medicine
		High-risk nature of some specialties like surgery or gynecology
		Uncertainty about the prognosis of the disease of individual patients
		Higher risk of admitting complicated patients referred from other centers.
	Factors related to the nature of the medical intervention	Uncertainties about some para-clinical test results
		Uncertainty about the outcomes of medical interventions.

**Table 4:** Examples of positive and negative DM

	Examples of DM
Negative DM	Exaggerating about the seriousness of the patient’s health condition to encourage them to continue treatment with another physician
	Exaggerating about the seriousness of the patient’s health condition reduces the patients’ and families’ expectations of the clinical outcomes and prevents the probability of future complaints, violence, or other negative consequences
	Refusal to admit patients who are more likely to sue their physician due to their personality or other characteristics such as their occupation (such as a lawyer).
	Avoiding risky and complex medical and surgical procedures
	Dissuading patients from continuing treatment by providing false information such as treatment by unskilled students, the slowness of the treatment process, etc.
	Unnecessary referral of patients to Tehran or provincial centers due to the lack of facilities
	Absence of physicians at the bedside of critical patients
	Refusal to admit high-risk, complicated, or referred patients
	Exposing patients to unnecessary interventions and refusing to address underlying problems to discourage them from continuing treatment
	Avoiding practicing medicine in unfamiliar regions due to lack of public trust and feeling of insecurity.
	Avoiding practicing in areas where, based on previous experience or evidence, violence against medical professionals is more likely.
	Avoiding practicing in areas where complaints from doctors are more likely.
	Refusal to admit patients such as celebrities, politicians, and journalists whose news is subject to significant public attention due to their social position.
	Wasting the patient’s time to transfer him/her to the next shift
	Filling up ICU beds with low-risk patients instead of high-risk patients to reduce the likelihood of lawsuits against physicians.
	Refraining from making explicit decisions for the patient and entrusting the decision to him with the justification of respecting the patient’s autonomy but with the aim of avoiding responsibilities and possible negative consequences.
	Leaving medical activities and focusing on other activities such as research or medical equipment trading
	Refusing to choose high-risk specialties to avoid complaints, stress or other types of challenges.
	Shifting the field of practice from high-risk medical procedures to less risky activities such as cosmetic measures.
Positive DM	Asking for multiple consultations to involve other colleagues in the treatment process
	Prescribing additional drugs to convince the patient about the importance of the actions taken.
	Requesting multiple unnecessary paraclinical procedures for the patient
	Obtaining acquittal from patients (in the hospital or even in the notary public office) in addition to informed consent
	Over-documentation of performed procedures
	Treatment of problems unrelated to the chief complaint of patients
	Using invasive para clinical procedures when it is possible to diagnose with physical examination or simple para clinical procedures
	Confusing patients with useless procedures when the physician is unable to make a correct diagnosis or treatment decision
	Obtaining multiple and unnecessary informed consent forms from patients
	Unnecessary ICU admissions
	Entering patients with no indication into screening programs
	Refusal of laboratories and para-clinical centers to provide a definitive answer and requesting rechecks due to fear of error
	Multiple patient visits or consultations
	Using medical interventions contrary to scientific standards to avoid complaints (like non-medically indicated C-sections due to the possibility of complications such as cerebral palsy)

**Table 5:** Strategies suggested by participants to prevent or reduce DM

Themes	Codes: Suggested Strategies to prevent or reduce DM
Suggested Strategies related to modification of medical complaints managing system	Consolidation of institutions for handling patient complaints in one organization and using standard methods for managing the complaints
	Strengthening the scientific aspects of the judgment in courts by increasing judges’ awareness of medical issues or seeking consultative opinions from physicians
	Conducting an initial review of complaints before summoning doctors to judicial courts to ensure the necessity of starting legal proceedings
	Developing and strengthening the process of handling valid complaints at the hospital or health facility level and preventing filing lawsuits in courts
	Dealing with complaints as much as possible at the level of hospitals or medical centers and preventing lawsuits in courts
	Excluding medical lawsuits from criminal liability
	Ensuring an acceptable level of respect for physicians during the complaints assessment process
	Clarifying judicial and disciplinary rules and the process of handling complaints against physicians
	Making decisions about physician practice based on each patient’s unique clinical condition rather than focusing on para-clinical data
	Developing the necessary sensitivity in legal and technical evaluations
	Dealing with complaints from physicians based on their commitment, duties and responsibilities and not just based on the results obtained.
	Strengthening a systematic view toward medical errors in complaint-handling organizations
Social strategies	Using all the available capacities, including the media to promote the health literacy level of the society
	Trying to reduce the circulation of false and pseudo-scientific information in society and social media.
	Correcting the false belief that referring patients to colleagues indicates incompetence
	Acquainting the community with their real rights to prevent unfounded complaints
	Promoting public trust in the self-regulatory system of the Medical Council to avoid referring cases to court.
	Trying to reduce social harassment against physicians and to maintain and promote public trust in the medical system
	Educating patients to demonstrate polite and peaceful behavior toward healthcare providers
	Removing the negative role of some lawyers that encourage people to file lawsuits against physicians
Organizational-managerial strategies	Strengthening and reforming reimbursement systems such as liability insurance for physicians
	Equipping hospitals, especially public hospitals and those located in provincial centers, to manage critically ill patients properly
	Providing sufficient information to patients and informing them about the possibility of complications
	Strengthening the necessary mechanisms to protect the right of physicians
	Improving the analytical power and decision-making ability of doctors through the improvement of education and evaluation methods, including turning memory-based questions into analytical questions.
	Integrating education related to consequences of DM and medical economics in the educational curriculum of medical students
	Offering education on communication skills to healthcare providers
	Installing appropriate monitoring systems to evaluate physicians’ practice and prescriptions
	Establishing a health economics committee in each hospital to monitor and guide physicians’ activities
	Enhancing the scientific capability of physicians to improve their self-confidence
	Placing more emphasis on the significant role of effective communication, accurate physical examination, history-taking, and clinical reasoning in the education of medical students instead of excessive use of para clinical services
	Management of patients visits in such a way that enough time can be allocated to each patient.
	Reducing the working hours of physicians to reduce the odds of error
	Strengthening the referral system to reduce the odds of error
	Improving medical education for correct medical management and decision-making
	Designing and developing medical information update systems
	Designing and conducting research activities to prove the ineffectiveness of excess interventions
	Inviting experienced clinical professors to seriously participate in teaching non-defensive medicine to medical students
	Developing national guidelines, institutionalizing their routine use by clinicians, and using them for formal adjudication by complaints handling agencies
	Increasing the motivation of physicians through revising medical tariffs and improving their financial prosperity
	Creating jobs and workplaces for doctors through reinforcing support systems
	Determining the permissible ceiling for para-clinical requests for different levels of doctors
	Holding clinical competence courses to make physicians familiar with their rights and responsibilities towards patients, and complaint-handling organizations
	Improving physicians’ professionalism through methods such as continuous education
	Considering serious punitive deterrent measures for physicians who repeatedly request excess para- clinical services
	Controlling the costs of excess para-clinical procedures by increasing the supervision of medical insurance
	Moving towards eliminating a direct financial relationship between physicians and patients
	Improving the educational system in terms of providing accurate and correct documentation training for performed procedures
	Accurate assessment of applicants to medical education courses to ensure that they do not have personality disorders
	Providing legal support for physicians through hiring lawyers in health centers
	Creating a balance between medical equipment in medical centers and society’s expectations from physicians
	Training members of complaints handling commissions to treat physicians respectfully
	Formulating treatment plans and standard procedures for each hospital proportional to its equipment and characteristics by experienced professors
	Providing more financial and legal support for physicians regarding admission and treatment of high-risk patients
	Providing grounds for cooperation and collaboration of all health policymakers and stakeholders in the Ministry of Health, Medical Council, Legal Medicine Organization, and Judiciary to address DM
	Research into underlying factors of the prevalence of DM in Iran and using other countries’ experiences to reduce DM
	Changing the payment system to physicians instead of payment based on type and number of interventions to a fixed payment

###  Underlying and contextual factors of DM

As per the participants’ responses (Table 3), the initial main category of findings pertains to the underlying and contextual factors of DM. These issues have been further classified into four primary themes, namely organizational-managerial factors, social factors, personal factors and factors associated with the nature of medicine and medical interventions.

Indeed, one of the factors linked to DM is the “insufficiency of scientific and practical skills,“ leading to the utilization of numerous para-clinical interventions in an attempt to avoid errors. When healthcare providers lack adequate scientific or practical training in a particular area, they might resort to unnecessary lab tests or imaging to mitigate the risk of errors, malpractice, and potential patient or companion complaints. One illustrative example that highlights this issue is the diagnosis of idiopathic facial nerve palsy, which can be differentiated from other cerebrospinal diseases accurately through a thorough physical examination. In such cases, para-clinical measures are unnecessary. However, some doctors opt to request a CT scan for diagnosis despite the availability of accurate physical examination findings.

In this context, one of the interviewees (No. 10) stated, “Physicians with lower experience and knowledge levels tend to request more para-clinical services to avoid complaints. Therefore, one of significant reasons for the prominence of this issue in the health system is the reduced involvement of senior faculty members [in patient care]. As a result, DM becomes more prevalent. Thus, the presence of professors with extensive knowledge and clinical skills plays a vital role in minimizing defensive practices [in university hospitals]”.

Another notable aspect concerning physician training was the “absence of local guidelines”. In participants opinion unavailability of approved local guidelines can adversely affects physicians’ self-assurance in admitting and treating high-risk patients, leading to both excessive precautionary measures and potential hesitancy in critical situations. But it is not only physicians who need guidelines but also patients need to have better understanding of their importance. As one interviewee (No. 38) emphasized, “Both the culture of physicians and patients must embrace guideline adherence to achieve optimal outcomes”.

The inadequate legal and professional support available to physicians can lead to a defensive approach in their practice. Even when a complaint lacks scientific and rational basis, and the physician is eventually acquitted after multiple appearances before review commissions, there exists no mechanism for restoring their dignity or compensating them for the damages incurred. Physicians often find themselves in situations where they must seek shift coverage from colleagues, cancel surgeries, or endure long commutes to appear before courts or disciplinary commissions of complaint-handling organizations. These requirements impose significant mental and financial burdens on the physicians. As highlighted by one of the participants (No. 22), “In my opinion, the primary layer of support for physicians should encompass comprehensive assistance, including insurance coverage and legal support. Presently, there are ongoing debates in our country concerning the possibility of sentencing physicians to prison, which is dangerous and can lead to DM. [To alleviate these concerns], it is crucial for colleagues to have appropriate insurance coverage, especially considering the increase in Diya (blood money in Islamic law). Having good insurance, provided by the health facility, would grant physicians peace of mind”.

Another significant factor contributing to the issue is the failure to allocate sufficient time for patient examination and clinical judgment. Consequently, multiple para-clinical interventions are often employed as a precautionary measure to avoid errors and malpractice. Furthermore, the shortage of medical equipment in less affluent centers is a critical concern. One of the participants, a manager with extensive experience in policymaking (No. 36), expressed her perspective on the matter: “DM is a prevalent practice within the medical field. The observation here is that DM is becoming more common in settings with limited equipment. When our residents graduate from well-equipped referral hospitals, they gain exposure to a wide range of procedures they can confidently perform. However, when they move to centers where such equipment is not available, they may find themselves less capable and more prone to adopting defensive practices”. Another participant said: “In my opinion, in settings with limited resources and inadequate equipment, physicians may resort to DM when faced with the need to perform invasive procedures. They tend to involve others by requesting numerous tests and consultations. [In sum] in environments where complaints are more frequent and resources, including equipment and workforce, are scarce, DM becomes even more prevalent”. According to the participants’ perspectives, other underlying factors of DM are associated with the patient complaint management system in the country. The experiences of being summoned to court and the stress faced by physicians due to unpleasant experiences of their colleagues with the trial process in courts or other complaint-handling organizations are among other factors contributing to an increased risk of defensive practices by physicians. In this regard, one of the participants (No. 1) said: “The older ones share their experiences with the younger ones. They recount how certain situations happened to them and emphasize that by requesting specific consultations or [prescribing or doing more medical] procedures, they could have avoided being summoned to a hearing session in court or facing a lawsuit from a patient’s family”. These experiences shared by colleagues contribute to stress among physicians, leading them to adopt DM practices. Another concern raised by the interviewees regarding the complaint-handling system is the arbitrary and non-specialist judgments made by complaint-handling organizations. One of the participants, a gynecologist with experience in management of healthcare facilities (No. 13) said: “During court proceedings, experts often question why we did not perform a Cesarean section. Unfortunately, there is no protocol in place to prevent such arbitrary questioning. Instead of adhering to a standardized protocol, these experts seem to act arbitrarily”.

Among the noticeable underlying causes and contextual factors identified by the participants of this study, social factors played a significant role. A key social factor contributing to DM was the low level of trust between physicians and their patients. As highlighted by one participant with substantial experience in health administration (No. 9), DM is inherently linked to the mutual trust between patients and physicians. In other words, the defensive behavior of physicians might stem from their lack of trust in patients, and this, in turn, can lead to a deterioration of patients’ trust in their doctors. When patients perceive a lack of trust from their physicians, it can seriously damage the crucial doctor-patient relationship, further exacerbating DM, he quoted: “One can never trust a person who did not trust him”. In essence, when there is a lack of trust between the physician and the patient, it leads to heightened concerns about potential complaints, ultimately leading to DM practices.

Another significant social factor contributing to DM is social violence and harassment directed towards physicians, which can harm their dignity. Additionally, patients’ expectations for and satisfaction with an abundance of paraclinical tests also play a role. At times, patients feel more content when physicians order newer and more tests, which can inadvertently promote defensive practices. One of the participants (No. 35) said: “In Iranian culture, there is a preference for numerous tests and imaging studies. It is perceived as a sign of greater attention being given to the patients’ needs and concerns”. Another interviewee (No. 1) similarly pointed out: “At times, there are situations where you may consider discharging a patient without a prescription. However, the patients often expect to receive a prescription, and interestingly, the more expensive the prescribed medication, the more they place their trust in you”.

Highlighting the negative portrayal of physicians in society and the role of mass and social media in shaping this perception is of utmost significance. Defensive practices among physicians stem not only from the fear of legal litigation but also from concerns about potential violence and harassment. Regarding the aggressive behavior of the patients, one of the professors (No. 35) said: “I notice that patients exhibit demanding behavior towards doctors, which goes beyond what is typically expected. While physicians have certain rights towards their patients, and vice versa, this behavior exceeds those bounds”.

Apprehensions regarding being accused of scientific and practical incompetence when referring patients to a colleague were another aspect influenced by the public culture. In this regard, one of the participants (No. 21) said: “Patients often fail to understand that when a physician refers them to another doctor, it doesn’t imply that the referring physician lacks knowledge or expertise. It is an act of compassion and the pursuit of a more accurate diagnosis. Unfortunately, this prevailing culture is not well understood by either the patients or the referring physicians. Consequently, I find myself hesitating to refer my patients, leading to the request for unnecessary actions”.

The increasing awareness of patients about their rights and their subsequent rise in complaints against physicians is another factor influenced by the prevailing public culture. One of the interviewees (No 1) said: “Previously, DM was more prevalent in large cities like Tehran [the capital city of Iran] and areas where patients were more aware of their rights. However, nowadays, this phenomenon is observed across various locations”. He continued: “One notable factor is the increased awareness of people regarding their rights, and this is an undeniable fact. In the past, patients may have viewed a doctor’s efforts as an act of goodwill, but nowadays, they say No! I have rights! [assert their rights more emphatically]. They [patients] have more awareness about their entitlements”. Another factor linked to the public culture was concerns about the irrational reaction of people to medical errors or complications, particularly in rural areas and small cities.

Personal characteristics of physicians can play a significant role in the practice of DM. Some of these characteristics include a lack of motivation for risk-taking, a cautious approach, and certain personality traits such as being obsessive or having low self-confidence. For instance, the poor motivation of physicians for risk-taking, partly due to unrealistic medical tariffs, was highlighted by one participant (No. 23): “These (low tariffs) are one of the factors; when there is no financial incentive and life [of physician] is challenging, why should you take on the responsibility of patients with a high probability of mortality, knowing they are very likely to file complaints?”. Consequently, in the absence of these motivations, physicians may lean towards negative DM, leading them to avoid admitting and treating high-risk patients. Alternatively, they might resort to positive DM by requesting several unnecessary para-clinical procedures to gain reassurance.

Another personal factor influencing DM is the personality traits of physicians, which can include low self-confidence, conservatism, obsessiveness, and an inability to manage pressures, among others. One of the interviewees (No. 24), who was also a health policymaker, mentioned: “Certain individuals (physicians) may exhibit more obsessive tendencies compared to others. For instance, a doctor conducting a physical examination might display heightened caution. This cautious nature may also extend to other aspects of their life and be reflected in their medical practice, where they may request an extensive list of lab tests and imaging studies that another physician might not request”. Another example is personality traits that include low levels of self-confidence leads to involving colleagues in the treatment process by seeking consultation and multiple para-clinical tests.

Some of the underlying and contextual factors reported by the study participants were related to the nature of specific medical interventions. These factors are associated with the complexity and high-risk nature of certain diseases and surgical procedures, leading to DM. Examples of such situations include Cesarean section in cases of placenta accreta, spinal cord or skull base tumors surgeries. When the intervention involves high-risk and complexity, physicians tend to become more cautious and may opt for defensive practices. Moreover, issues like uncertainty in the results of para-clinical tests, negative outcomes of certain medical interventions, and poor prognosis of diseases are among other factors influencing physicians’ defensive approach.

### Examples of defensive medicine

The second primary category of findings from this study, as depicted in Table 4, pertains to various ways in which physicians employ DM. These examples and practices mentioned during interviews are categorized into two main groups: positive DM and negative DM. In this instance, we have employed the well-established and widely recognized classic positive-negative distinction, which has been extensively utilized in the existing literature. Positive DM refers to interventions that are not medically necessary but are used as additional diagnostic or therapeutic measures (such as tests, procedures, or office visits). The main motivation underlying these actions is to protect the doctor from potential complaints or objections from patients or their companions, as well as criticism from colleagues. One of the prominent examples of positive DM mentioned by all participant groups, including policymakers, managers, and physicians, is the practice of requesting multiple consultations to involve other colleagues in the treatment process. The purpose of this practice is to implicate other physicians in the decision-making process, providing the doctor with an excuse to defend themselves in case complications arise, the patient files a complaint, or to prolong the transfer of the patient to the next shift.

One of the participants (No. 17) with management experience provided an illustrative example of positive DM, stating, “For instance, you may plan to perform a patient’s surgery, but the anesthesiologist declines to proceed and insists on a consultation. The next day, when the consultation report is ready, another anesthesiologist is asked to proceed with the patient’s admission”. Another instance of positive DM, as viewed by managers and policymakers, involves the request for multiple para-clinical interventions, including unnecessary lab tests and imaging. One of the professors (No. 3) raised ethical concerns about this practice, stating, “When a physician imposes an unnecessary intervention or procedure on the patient, it absolves the physician of potential problems that might arise. However, this can place a heavy burden on the patient. From an ethical perspective, it is not acceptable to impose a significant financial burden on patients solely to protect ourselves or prevent them from raising complaints”.

Negative DM refers to the practice of physicians refraining from performing necessary interventions for patients, including the admission of high-risk patients, or undertaking high-risk procedures, in an effort to avoid potential complaints or harm in the future, such as violence directed at the physician by patients or companions. Several examples of negative DM were cited by all three groups of this study participants, including physicians specializing in field with more high-risk patients. Participant No. 22 highlighted one situation, stating, “In emergency cases, physicians diligently fulfill their duties to the best of their ability. However, when it comes to elective cases, there is a tendency to avoid admitting high-risk patients, those with a substantial risk of mortality and morbidity”. Another example of negative DM involves exaggerating the patient’s health condition, typically done to prepare patients and their companions for the worst-case scenario to manage their expectations of the treatment outcome and prevent complaints if the treatment is not successful. A professor of surgery (No. 40) provided insight into this practice, saying, “Sometimes, as physicians, we tend to unnecessarily frighten patients and their families when explaining potential complications, just to absolve ourselves and ensure they won’t file complaints against the doctor or the medical team in the future. We might magnify a complication that occurs in only 1% of cases and could be easily explained, but we present it as something bigger [more severe and frequent]”.

###  Potential strategies to mitigate and prevent DM

The potential strategies to mitigate and prevent DM emerged as the third main category of results. As per the participants, one of the most crucial strategies to mitigate DM is related to the management of complaints. They suggested employing judges who are familiar with the medical context or encouraging them to seek advice from physicians before making decisions. By doing so, physicians can be reassured that complaints will be handled according to scientific principles and sound judgments will prevail. Participant No. 9 expressed this notion, stating, “Appointing knowledgeable judges is essential. It’s not just about being an expert; [physician] experts in the Medical Council [who judge cases] should also possess some understanding of the judgment process and also place themselves in the physician’s shoes”. Another strategy pertaining to the complaint handling system involves assessing the physician’s performance based on the patient’s clinical condition rather than solely focusing on para-clinical interventions. Moreover, participants emphasized the significance of showing respect to physicians during the trial process. Being frustrated from multiple organizations who are dealing with patients’ complaints such as courts, Forensic (Legal) Medicine Organizations, Medical Council, Governmental Discretionary Punishments Organization (GDPO) and regional headquarters of the Ministry of Health (Medical Universities) and suggested assigning one organization responsible for managing patients’ complaints, along with implementing standardized procedures to ensure fair judgment and respectful treatment of physicians.

Another set of suggested strategies for addressing DM focuses on the social aspects of this phenomenon. One such strategy involves promoting public trust in the medical system through various means, including utilizing the media. A health policymaker who participated in the interviews emphasized that “the rate of patients’ complaints and DM practice largely depend on physicians’ behavior and their trustworthiness in the eyes of patients. Their trust, I mean the patients’ trust in the medical system depends on the physician’s behavior and practice”. Building trust in the medical system can be achieved by removing factors that negatively affect it, such as discouraging lawyers who encourage patients to make complaints and enhancing public trust in self-regulatory systems of medicine.

Modifying the expectations of the general population and patients and making such demands realistic is considered another effective strategy to reduce and prevent DM. To achieve this, the participants suggestion was improving the health literacy level of society, as highlighted by one of the interviewees (No 27): “When people lack sufficient education about medical matters, they may, for instance, mistakenly think they need to consult 10 subspecialists for a simple common cold. Even if the first doctor advises rest for 2 days, plenty of fluids, and reassures that antibiotics are not necessary, some individuals may still doubt this advice and insist on receiving an antibiotic injection”. Some participants proposed strategies to modify patients’ demands, including efforts to correct the misconception that prescribing more drugs and tests indicates the physician’s higher competence.

Another crucial strategy for enhancing the public culture is to monitor the media’s activities, as it serves as the most popular communication tool for upholding the dignity of the medical professionals. Participant No. 6 emphasized: “The rules should be modified to prevent individuals without relevant education or medical expertise from making comments on the medical society, particularly in media outlets such as radio, TV, newspapers, and different tribunals like the parliament. Medical issues should be handled and addressed exclusively by qualified medical experts”.

Participants in this study highlighted another set of approaches to reduce or prevent DM, termed organizational-managerial strategies. One aspect of these strategies is related to the education and training of physicians, with a focus on enhancing effective patient-physician communication, emphasizing physical examination, history-taking, and clinical reasoning, rather than over-relying on para-clinical services. In this regard, one of the interviewees (No. 7) said: “At times, the occurrence of this phenomenon can be attributed to incorrect or insufficient training provided to medical students or residents. [Although] there could be several reasons behind this issue, [but] we can see that it is prevalent in all university hospitals”. Improving the training provided to physicians to enhance their clinical judgment, patient management skills and decision-making abilities was identified as another educational strategy. One of the interviewees (No. 38) mentioned the quality of education: “I believe the most crucial solution is to establish guidelines, enhance the quality of medical equipment (rather than focusing solely on quantity, as we already have sufficient quantity), and provide proper and comprehensive education to our doctors”. Other strategies raised by the interviewees were to improve the referral system and to teach communication skills to health care providers.

Another strategy related to health system policymaking involved promoting and modifying the reimbursement systems of liability insurance for physicians. The presence of such reimbursement systems gives physicians the confidence to admit high-risk patients. Participant No 22 emphasized the importance of supporting residents with such insurance coverage and stated “It is particularly crucial to ensure that our residents have proper insurance coverage. All of them should be insured by the universities”. Other suggested strategies were implementing a proper supervisory system to oversee the prescriptions and activities of physicians, formulating local and national guidelines and institutionalizing their routine use by physicians, with court judgments based on these guidelines, establishing job security for physicians by reinforcing supporting systems, providing financial and legal support for physicians in admitting and treating high-risk patients and initiating a collaborative set of actions by the related stakeholders to address the issue of DM, as the participant No mentioned: “I believe our system should be better coordinated, with collaboration among our courts, Medical Council, insurance companies, hospitals, and Ministry”.

## Discussion

The present study revealed a spectrum of suggested underlying and contextual factors, examples, and preventive strategies for addressing DM. Study participants highlighted some issues which in their views could be counted as potential causes of DM. While such causes could be significantly diverse in the viewpoint of various stakeholders, including healthcare professionals, patients, and health policymakers, ascertaining causality in the realm of social science poses even more significant fundamental challenges, as there is no objective means to definitively determine the truth. Therefore, such issues should be considered as causes that were attributed by study participants. Social scientists may also hold differing interpretations of the dynamics within medical practice. Consequently, attributing causality within the realm of social science can be highly problematic, and there is no objective way to determine what is really happening. In this area what causes or generates DM can be understood in diverse ways, depending on various social ontology and overarching social theory. Therefore, we preferred to use the language of underlying and contextual factors instead of causes which directly implies a causality relation. As an example, while exploring the causes of DM, two contrasting social theories could emerge. One theory places physicians’ decision as the pivotal factor, suggesting that they are the ultimate deciders shaping defensive practices. The decision-making process they encounter may influence their choices, but the responsibility lies with the physicians. In contrast, another theory emphasizes the decision architecture of the situations in which physicians make decisions. This second theoretical point of view reduces the individual physicians’ agency. As we can see, these theories offer valid perspectives from a social science standpoint, and it is unrealistic to expect any study to definitively resolve this tension between macro-level (structural, collective) and micro-level (individual, personal) explanations of causes that could generate DM.

Regarding the causes, the present study mentioned underlying factors like concerns about complaints, physicians’ fear of the trial litigation process due to their or their colleagues’ unpleasant experiences, and lack of proper liability insurance, which have been previously discussed in other studies [23]. This finding was also reported in a retrospective 12-year analysis of medical malpractice claims of the Taiwan civil courts. This study found that medical malpractice lawsuits increased physicians’ stress and reduced their job satisfaction, which could result in defensive practices to prevent complaints [24].

However, some underlying factors of DM found in this study were less addressed in the literature. These include refusal of insurance companies to provide full liability insurance coverage for physicians, physicians’ concerns about being insulted or harassed by patients or their companions, fears of being humiliated or marginalized by colleagues, concerns about their dignity being violated, doubts about their competence, anxieties over losing their social and scientific status, lack of sufficient scientific and practical skills, shortages of equipment and facilities in less affluent centers compared to referral university hospitals where doctors are trained and skilled, multiplicity of complaint-handling organizations, an increasing familiarity of patients with their rights leading to more medical complaints, projecting a negative image of physicians in society, low self-confidence among some doctors, and the conservative or obsessive personality of some physicians.

Based on the insights provided by study participants, one of the primary sources of fear among physicians regarding legal litigations and complaints seems to be connected to concerns about their dignity. Consequently, the idea of dignity restoration emerges as a promising approach, given that accusations of improper practice can have enduring consequences on an individual’s life and professional journey, even after being legally exonerated. In such scenarios, physicians become more susceptible to stigmatization, leading to potential marginalization within the medical community. Within this context, two types of stigmas can be discerned: legal stigma and moral stigma. Legal stigma often accompanies moral stigma, but not vice versa. Carrying the weight of legal stigma, if it persists, can be immensely burdensome. However, even in cases where legal stigma does not endure, there remains the possibility of moral stigma lingering, affecting an individual’s reputation and self-perception.

Another contextual factor that exacerbates DM in Iran’s health system is the fragmented approach to handling medical errors or ethical misconduct. The current system lacks integration, resulting in multiple organizations being involved in addressing medical malpractice cases, including courts, the Medical Council, the Ministry of Health, and the Governmental Discretionary Punishments Organization (GDPO). This lack of a cohesive and streamlined system can lead to confusion and inefficiencies, further contributing to the practice of DM among healthcare professionals. Addressing this issue and establishing a more coordinated and transparent framework for handling medical errors is essential to alleviate the burden of defensive practices and improve the overall quality of healthcare in the country.

DM encompasses distinct aspects, including social and structural elements, and should not be solely attributed to physicians’ behavior. Several participants argued that it is a systematic problem, and the healthcare system needs restructuring to ensure physicians are not compelled to resort to DM as a means of self-protection or to avoid consequences related to objections and complaints from patients and their companions. Multiple factors, such as policymaking, laws, and managerial and administrative influences, contribute to the development and practice of DM. Therefore, it is essential to devise proper policies and enact necessary laws and regulations to garner support from relevant organizations and authorities for standard medical interventions performed by physicians and to prevent the occurrence of DM. Successful reduction of DM requires collaborative efforts across different sectors. In this regard, one of the most crucial interventions suggested as a strategy in various parts of the world is the decriminalization of medical errors [11, 25].

One of the factors related to the management of health centers and health system policymaking is “the lack of an efficient legal and coherent system to support physicians“. The concern for safeguarding the rights of medical professionals led to the enactment of the Bill of Rights of Iranian Medical Professionals by the Supreme Council of Iran Medical Council in 2021. This Bill addresses various aspects of the rights of medical professionals, including facilitating access to an efficient support system. Article 9 of the Bill states that “Members of the medical profession have the right to access the consultation system and legal supports of the Medical Council of Iran if their rights, as mentioned in the Charter, have been violated. The Medical Council must establish an appropriate mechanism to guide its members and facilitate their access to legal consultation services. Moreover, the Medical Council is obligated to defend the professional and personal dignity and security of its members when it becomes evident that they are facing prosecution, summons, etc. due to or as a result of carrying out their professional duties correctly and in accordance with scientific, technical, and ethical standards” [26]. Legal support would be particularly valuable, especially in reducing the prevalence of negative DM practices.

Promoting a protective and supportive umbrella of comprehensive liability insurance, which includes removing the time limit for reimbursement in professional liability insurance, increasing insurance coverage, and extending the time limit after medical interventions, was another strategy proposed by the interviewees. Currently, liability insurance is bound to reimburse the costs of any medical intervention within 4 years after the procedure. However, it refrains from reimbursement if the physician is sentenced to pay indemnity after this time for any reason, including the late complications of some medical errors and lengthy trials. Moreover, increasing the amount of insurance commitment alleviates physicians’ concerns and encourages them to practice without worrying about patients’ complaints and the financial consequences that may follow.

Another significant contextual issue, related to the legal system, contributing to the prevalence of DM in Iran’s health system is the quality and specificity of medical laws. Currently, these laws are inadequately developed and in need of updating. The existing legal framework may not sufficiently address the complexities and nuances of modern healthcare practice, leaving healthcare professionals uncertain about their liabilities and legal protections. Similar to medical guidelines, the lack of clear and up-to-date laws can create an atmosphere of uncertainty, prompting healthcare providers to adopt defensive practices as a precautionary measure to protect themselves from potential legal challenges. In addition to the content pf laws, our findings indicate the necessity for clarifying the rules and the process of handling patients’ complaints in courts of law.

Another example of cultural issues that significantly influence the prevalence of DM is the prevailing misconception in society that doctors with greater knowledge and skills tend to prescribe more drugs and para-clinical procedures. This misguided belief inadvertently drives healthcare providers towards adopting a positive defensive approach. Under the weight of this misconception, physicians may feel compelled to overprescribe medications and diagnostic tests as a protective measure. The fear of being perceived as less knowledgeable or competent by patients or colleagues pushes them to take a more cautious approach, even when it may not be clinically necessary.

Physicians often find themselves facing criticism, objections from patients and their families, and even social and media harassment if treatment objectives are not met. Additionally, they may have to attend different commissions and hearing sessions to defend themselves and explain the reasons behind their medical decisions. Furthermore, the tariffs for high-risk surgeries are often similar to those of simple surgeries or aesthetic procedures, despite the fact that high-risk surgeries are much more complex, take several hours to perform, carry a higher risk of complications, and result in significant stress for the physician during and after the procedure. As a result, physicians may choose to avoid admitting high-risk patients or performing such surgeries to preempt future problems and complaints. Based on our findings, the social structure of the society, the prevailing culture in clinical environments, the level of public trust in the medical society, and the social capital of the society all influence the DM behavior of physicians. In addition, unrealistic tariffs and the absence of a well-implemented referral system have led to an increase in working hours and patient visits for some physicians, leading to fatigue and burnout. Consequently, they may end up taking incomplete medical histories from patients and resorting to defensive practice. This problem is less prevalent in countries with better economic conditions and where physicians’ income is satisfactory.

While it may be challenging to ascertain the precise empirical approach for achieving this goal, the notion of fostering trust in physicians, nurturing patient-physician trust, and bolstering overall trust in the healthcare system is highly commendable. Trust plays a pivotal role in shaping the dynamics between physicians and patients, either mitigating or exacerbating litigious tendencies in their interactions. In essence, viewing the physician-patient relationship through a litigation lens is often associated with diminished trust, while a heightened level of trust between them tends to reduce the inclination towards litigation. Although the exact psychological mechanisms driving this phenomenon remain unclear, it aligns with existing research on litigious behavior and resonates with the experiences of seasoned lawyers well-versed in handling legal matters. Ultimately, cultivating an environment of trust has the potential to positively influence the nature of physician-patient interactions and promote a more harmonious and collaborative approach to healthcare. While the path to achieving this may be multifaceted, acknowledging the significance of trust represents a crucial step in fostering a constructive and patient-centric healthcare landscape.

The demand and satisfaction of patients and their dependents with multiple paraclinical tests sometimes lead physicians to prescribe unnecessary treatments. In some cases, physicians respond positively to patients’ demands and prescribe drugs and tests without scientific indications to appease their patients and prevent objections and complaints. The issue of defensive practice due to patients’ demands was also addressed in a study conducted in the United States in 2017. The survey found that 20.6% of medical care provided in the United States was unnecessary, and requests or pressure from patients was identified as one of the two significant reasons for unnecessary care [13]. These unnecessary treatments may result in unwanted side effects. For instance “in a patient with an infection a physician practicing DM may prolong antibiotic duration, prescribe unnecessary broad-spectrum antibiotics or combinations of agents, or prescribe unnecessary antibiotic treatments which may contribute to the alarming spread of antibiotic resistance” [27].

In recent years, there has been a rise in the education and literacy level of people, and their awareness of their social rights has also improved. This progress has led to an increase in patients’ demands and complaints from healthcare providers, causing physicians to worry about the consequences of such complaints and practice defensively. While the improved health literacy of people and their awareness of their rights are significant achievements, it is crucial to foster an appropriate culture and provide proper education and training to prevent unfounded complaints about physicians. While this social change is not properly understood and responded to by medical professionals.

Despite the study participants suggesting that increasing health literacy among the general public could be a potential strategy to address DM, a crucial point to consider is the complexity of gauging the required level and types of health literacy necessary for patients to fully comprehend their physicians’ actions. In essence, much of what physicians do involves intricate clinical judgment that may not be entirely understandable to non-physicians. The inherent gap between the knowledge of physicians and patients can best be bridged by fostering trust. Trust plays a pivotal role in filling the void of understanding, preventing the emergence of baseless hypotheses, presumptions, or assumptions that might question the physician’s integrity, intentions, or the healthcare system as a whole. While enhancing health and science literacy is undoubtedly beneficial, its effectiveness in reducing DM remains uncertain and requires further exploration. While the study participants’ perspective on the role of health literacy level may be subject to challenge and evolution over time, being aware of this prevailing sentiment among physicians enables researchers and other stakeholders engaging with them to delve further into the implications of their proposals. It also encourages a deeper reflection on the assumptions surrounding the potential achievements of health literacy in healthcare.

Despite the major social changes, the prevailing culture in the Iranian medical system is still paternalistic, and establishing a patient-centered culture remains a distant goal. This approach in the physician-patient relationship lowers patients’ participation in medical decision-making processes and paves the ground for misunderstandings and subsequent medical complaints, perpetuating a vicious cycle. On the other hand, highlighting the negative portrayal of physicians in society and acknowledging the influence of mass and social media in shaping this perception is of utmost importance. In an atmosphere of widespread distrust, it is essential to recognize that defensive practices among physicians, driven by the fear of litigation, can be seen as a natural response.

Despite the study participants’ suggestion to limit non-professionals’ freedom to discuss medical practice in mass media as a measure to enhance social trust in medical professionals and reduce DM, this perspective could face opposition. While we can attribute such suggestions to the paternalistic approach of the particular participant, the non-liberal political system of the country, or to the paternalistic culture of medical practice in Iran, a significant concern is that such restrictions might actually erode trust in medical experts instead of reinforcing it. The reason is that when only approved experts are allowed to speak, the credibility of their opinions relies heavily on the endorsing institution. If these institutions lack trustworthiness, the experts’ credibility may be called into question, perpetuating a cycle of mistrust. Moreover, the feasibility of implementing such restrictions in the digital age and outside certain contexts, like Iran, appears highly implausible, particularly in liberal democratic societies. Additionally, the notion of allowing only properly qualified experts to publicly comment on medical practice raises the fundamental question of how one defines “properly qualified“. If designation as a qualified expert is solely dictated by medical authorities, it may lead to the imposition of an official message, which often hampers efforts to increase trust.

The problems related to the physician-patient relationship are not limited to personal relationships and have social and external manifestations and may reduce the self-confidence of the physicians and foster an obsessive caution toward prescribing diagnostic or treatment interventions. Participants in our study emphasized the significance of comprehensive practical and scientific training for medical practitioners, which plays a crucial role in influencing physicians’ self-confidence and, subsequently, impacting defensive medical practices. However, it is essential to acknowledge that clinical judgment is a multifaceted issue that often develops over years of experience. Some other study participants further supported this notion by mentioning that senior physicians tend to be less inclined towards defensive practices.

It should be noted that all of the considerations that physicians apply to protect themselves against patients’ complaints collectively known as defensive practice are not necessarily unethical. For example, measures like careful documentation of interventions, especially when there is a risk of future complaints, receiving consultation on risk management, and seeking legal consultation in the hospital if done based on defined standards, are not unethical [13]. They are considered problematic when diagnostic and therapeutic interventions have no benefits for the patients and are done solely for defensive intentions. As discussed earlier, these interventions may carry a substantial risk for patients and damage the health system. The nature of clinical judgment being case-oriented adds complexity to determining the correctness of medical practice, especially in complex cases. As a result, ethically evaluating medical interventions and practices based on defensive motivations becomes far from straightforward. Objective determination of defensive practices faces challenges as it can be highly idiosyncratic, varying from case to case, making it difficult to arrive at definitive conclusions. As a result, obtaining a comprehensive understanding of the prevalence of such practices often relies on self-reports provided by physicians.

Regarding the strategies to reduce or prevent defensive practice, the present study proposed several strategies, some of which have been reported in earlier studies, such as the need to modify liability insurance and the reimbursement system. However, the present study also revealed several strategies to control defensive practice that have been less reported before. Another strategy proposed by the interviewees was to fix the system of handling complaints in order to reduce the number of complaint-handling organizations. According to them, the presence of different organizations causes wastage of physicians’ time and energy during different stages of the trial, disturbs their peace of mind and self-confidence, reduces their willingness to take risks in performing high-risk procedures, and discourages medical interventions, particularly complex surgeries. This situation leads to increased referrals to other physicians or healthcare centers, higher patient costs, and more harm to the patient. The recommendation is to develop and expand complaint-handling centers in hospitals to prevent filing lawsuits in disciplinary and legal organizations. This way, the primary evaluation of complaints can be done in the hospitals, and the necessity of summoning the physician to the complaint-handling organization can be ascertained before summoning.

Another suggestion was to correct the public misconception that patient referral indicates incompetence of the physician. While in such circumstances doctors usually refer the patients to an experienced colleague when find their ability insufficient for treating the patient in order to prevent damage to the patient. In the presence of such misconceptions, physicians might feel more comfortable to e.g., perform multiple paraclinical interventions.

Formulating specific standards such as national guidelines, institutionalizing their routine use by physicians, and making formal judgments based on them in complaint-handling organizations are other strategies aimed at preventing DM practice. When physicians adhere to diagnostic-treatment interventions in line with the guidelines, they fulfill their duties towards patients and have a strong defense if any complaints are filed. Consequently, there will be a reduction in defensive practice driven by concerns about patient complaints [27]. Such standards could be around issues such as reducing the number of patients or the working hours of physicians which can improve their concentration and reduces the odds of error and complaints and thus resolving their concerns about complaints and legal problems and decreasing their interest in defensive practice. Or considering DM as an issue while setting tariffs. Since unrealistic tariffs, and failure to implement the referral system has increased the working hours and the number of patient visits for some physicians, which in turn results in their fatigue and burnout, taking an incomplete medical history from the patients, and driving them toward defensive practice. This issue is less of a problem in countries where there are no important economic problems and the physicians’ income is acceptable.

DM extends beyond the realm of medical interventions on patients. According to the findings of the present study, defensive motivations not only alter physicians’ approach to medical interventions but also influence their choice of specialty and field of activity at a higher level. As a result, general physicians are less inclined to select specialty and subspecialty fields dealing with high-risk patients, leading to potential harm to this vulnerable group of patients. Furthermore, this defensive approach can manifest as routines and practical standards in clinical environments. For instance, parents’ demands for a brain CT scan in children with head trauma, lacking clinical necessity, have been considered an indication for a CT scan in emergency medicine reference books. In the ethical evaluation of DM, it should be noted that, in certain cases, this approach can be seen as a legitimate defense. Nevertheless, the prevalence of the DM approach in scientific texts underscores its wide impact on medicine. Therefore, it is essential to carefully assess medical education resources to ensure that such an approach is not imparted to medical students through medical texts. Simultaneously, increasing societal awareness regarding the negative consequences of inappropriate demands on doctors that drive them towards DM is crucial.

Considering the valuable insights gathered from a substantial number of interviewees who possess policymaking and administrative experience within the country’s health system and who are generally well-versed in medical malpractice laws and the medical litigation process, including individuals directly involved in the litigation process, the results of this study offer a strong foundation for informing policy decisions within the healthcare system. However, we acknowledge the complexity of the DM phenomenon, and there may be aspects that our study did not fully uncover. Considering the need for a multifaceted approach in policymaking, we encourage policymakers to complement the findings of this study with other available evidence to address any potential limitations and to gain a more comprehensive understanding of the policymaking process related to DM and can make well-informed decisions to effectively address the challenges posed by DM in the healthcare system”.

While the primary aim of this qualitative study was to increase comprehension and stimulate discussions about DM, it is essential to address the fact that some policy suggestions were made based on issues raised by the study participants. There are policy recommendations rather than definitive solutions to the complex and heterogeneous problem of DM, which emerged naturally from the data collected during the study. Heterogeneity could be a significant factor that complicates making macro-level claims such as policy suggestions. The study participants, being stakeholders with firsthand experience in the field, provided insights that led to the identification of potential areas for improvement to manage DM. It is crucial to acknowledge that these policy suggestions should be considered exploratory rather than conclusive. They may serve as starting points for further research and analysis. Additionally, policymakers and stakeholders should approach these recommendations with caution, understanding the limitations of the study and the need for more extensive research and empirical evidence to support any policy changes.

## Conclusion

DM is a significant and multi-dimensional phenomenon that profoundly impacts the health system, medical professionals, and patients. Considering a medical intervention or procedure as defensive practice hinges on the presence of self-protection motives. To fully grasp the various aspects of this phenomenon, one must carefully examine the complex structures, underlying factors, and contextual elements that contribute to it. Additionally, the examples and methods of defensive practice may differ based on social, cultural, legal, and professional backgrounds. Likewise, the strategies to reduce and prevent DM are intricately linked to the complexities and multidimensionality of this phenomenon. Consequently, a set of strategies at different personal, organizational, and macro levels might be proposed to effectively mitigate it.

While the results of this study offer a solid foundation for informing policy decisions within the healthcare system and include some explanatory policy suggestions, we encourage policymakers to complement the findings of this study with other available evidence to address any potential limitations and to gain a more comprehensive understanding of the policymaking process related to DM.

### Study limitations

All interviews were exclusively conducted in Tehran, Iran’s capital city, which could limit the representation of views and perspectives from professionals and policymakers in other provinces of Iran. While acknowledging this geographical limitation, we think that this constraint does not heavily impact the results to be generalizable to the whole Iranian context, given that all physicians in Iran are obligated to serve a period of work in remote areas. This mandatory service helps in garnering a more comprehensive understanding of diverse healthcare challenges and practices across the country and could be projected in their responses in the interviews. Furthermore, most of the interviewees had management and policymaking experiences and exhibited substantial proficiency and held ample experience and information at the national level. Their expertise ensured a thorough examination of prevailing issues within the Iranian health system. However, the limitation of the exclusiveness of the interviews with experts in Iran still remains. To further enhance the study’s comprehensiveness, future research could endeavor to include a broader representation of professionals from different international perspectives. This would offer a more comprehensive and holistic understanding of the subject matter and enrich the overall implications of the study’s findings.

### Supplementary Information

**Additional file 1.** Interview Guide.

## Data Availability

The datasets generated and/or analyzed during the current study are not publicly available due to ethical and privacy issues but are available from the corresponding author on reasonable request.
