# Supplementary material for: Exploring defensive medicine: examples, underlying and contextual factors, and potential strategies - a qualitative study
Source: BMC Med Ethics. 2023 Oct 10;24:82. doi: 10.1186/s12910-023-00949-2 (PMC10563204; doi:10.1186/s12910-023-00949-2)
Supplement: Supplementary file 1 — Additional file 1. Interview Guide. [file 12910_2023_949_MOESM1_ESM.doc]

Interview Guide

Examples, Underlying Causes, and Contextual Factors of Defensive Medicine and Possible Confronting Strategies: A Qualitative Study

Prior to the interview:

Send an email to the selected participant and introduce yourself and give a brief explanation of the study and why the investigators selected them to be invited as an interviewee and ask for a time of around 90 minutes for the interview. Attach a file consisting of a summary of the project to the email.

In case of non-response, send a reminder email and emphasize the importance of the interview.

In case of a negative response thank the invitees and politely ask if they can explain the reason for the rejection.

In case of a positive response, set a time for the interview and confirm the meeting time by sending a reminder email or SMS two days before the interview.

Ensure your recording instrument before the interview.

Try to be on time and attend the meeting 15 minutes before the time of the interview. Consider the traffic situation and other factors that might interfere with your timing.

Study title: Examples, underlying causes, and contextual factors of defensive medicine and possible confronting strategies: a qualitative study

Participant number:

Participant group (specialty):

Interview date:

Duration of the interview:

To start the interview:

- Ask for consent to turn the voice recorder on. Assure the participants about the confidentiality of the gathered data.
- After turning on the recorder, introduce yourself, the interviewee, and the date of the interview, and seek the interviewee’s consent to participate in the study and interviews.
- Explain the study’s objectives and justification, briefly explaining the background and defensive medicine definition and introducing the study’s team.
- Start questions one by one as below.
- During the interview, try not to interrupt the interviewee. If the discussion goes out of the topic, try to draw back the interviewee’s attention to the study’s objectives.
- In case the interviewees do not understand the question truly, give them examples of what is already said in literature or previous interviews.
- At the end of the interview, give a brief explanation about the next steps, the transcription of interviews, and the time that the research team keeps the recorded files and ask participants to ensure they have any other comments or suggestions.
- Prepare a visit card with your contact details and ask the participants to share any other issues, thoughts, or ideas with you through email.

The open-ended question would be asked at the beginning of the

interviews to help the participants freely share their perspectives, here are 3 main general questions and the probing questions that could be asked to deepen the answers:

1. How do you describe your understanding and experience of defensive medicine in Iran?

- Do you agree with the provided definition?
- Do you have any comments or recommendations to complete the given definition?
- Which methods or examples of defensive medical practices could be found in Iran?
- How do you explain these examples of defensive medicine, especially in your field?
- In your opinion, to what extent is Iranian medicine facing this phenomenon?
- In your opinion, which medical groups are more likely to turn to defensive medicine?
- What solutions do you suggest for controlling and reducing defensive medicine in Iran?

1. Which factors and underlying causes (social and contextual factors, national policies, organizational policies, health system policies, etc.) and contexts lead to the existence of defensive practices in Iran’s healthcare system?

- How do you describe the mechanism of shaping defensive medicine by these factors?
- In your opinion, which contextual factors may accelerate or aggravate defensive medicine in Iran?

1. Do you think defensive medicine is morally justified?

Why or why not?

- Please justify your point of view based on ethical standards like values, principles, rules, and virtues and explain your moral judgment.
- What do think about the patients' perspectives on this issue What would be their reaction if they realized that there are defensive motivations behind some medical intervention?
